# Spatial variation of perfusion MRI reflects cognitive decline in mild cognitive impairment and early dementia

**DOI:** 10.1038/s41598-021-02313-z

**Published:** 2021-12-02

**Authors:** Catherine A. Morgan, Tracy R. Melzer, Reece P. Roberts, Kristina Wiebels, Henk J. M. M. Mutsaerts, Meg J. Spriggs, John C. Dalrymple-Alford, Tim J. Anderson, Nicholas J. Cutfield, Gerard Deib, Josef Pfeuffer, Donna Rose Addis, Ian J. Kirk, Lynette J. Tippett

**Affiliations:** 1 School of Psychology and Centre for Brain Research, The University of Auckland, Building 302, Level 2, 23 Symonds Street, Private Bag 92019, Auckland, 1142 New Zealand; 2 Brain Research New Zealand—Rangahau Roro Aotearoa, Centre of Research Excellence, Auckland, New Zealand; 3 Centre for Advanced MRI, Auckland UniServices Limited, Auckland, New Zealand; 4 Department of Medicine, University of Otago, Christchurch, New Zealand; 5 NZ Brain Research Institute, Christchurch, New Zealand; 6 School of Psychology, Speech and Hearing, University of Canterbury, Christchurch, New Zealand; 7 Department of Radiology and Nuclear Medicine, Amsterdam University Medical Center, Location VUmc, Amsterdam, The Netherlands; 8 Department of Radiology and Nuclear Medicine, University Hospital Ghent, Ghent, Belgium; 9 Centre for Psychedelic Research, Division of Brain Sciences, Imperial College London, London, UK; 10 Department of Medicine, University of Otago, Dunedin, New Zealand; 11 Department of Neuroradiology, West Virginia University, Morgantown, WV USA; 12 Siemens Healthcare, Application Development, Erlangen, Germany; 13 Rotman Research Institute, Baycrest Health Sciences, Toronto, Canada; 14 Department of Psychology, University of Toronto, Toronto, Canada

**Keywords:** Cognitive ageing, Alzheimer's disease, Dementia, Biomarkers

## Abstract

Cerebral blood flow (CBF) measured with arterial spin labelling (ASL) magnetic resonance imaging (MRI) reflects cerebral perfusion, related to metabolism, and arterial transit time (ATT), related to vascular health. Our aim was to investigate the spatial coefficient of variation (sCoV) of CBF maps as a surrogate for ATT, in volunteers meeting criteria for subjective cognitive decline (SCD), amnestic mild cognitive impairment (MCI) and probable Alzheimer’s dementia (AD). Whole-brain pseudo continuous ASL MRI was performed at 3 T in 122 participants (controls = 20, SCD = 44, MCI = 45 and AD = 13) across three sites in New Zealand. From CBF maps that included all grey matter, sCoV progressively increased across each group with increased cognitive deficit. A similar overall trend was found when examining sCoV solely in the temporal lobe. We conclude that sCoV, a simple to compute imaging metric derived from ASL MRI, is sensitive to varying degrees of cognitive changes and supports the view that vascular health contributes to cognitive decline associated with Alzheimer’s disease.

## Introduction

There are approximately 50 million people currently living with dementia worldwide, a number set to increase three-fold by 2050^1^. Alzheimer’s disease (AD) is the primary cause of dementia, accounting for up to 70% of all cases^2^. The AT(N) framework^3^, a biological construct of AD, recommends measuring beta-amyloid deposition (A) pathologic tau (T), and neurodegenerative changes (N) as in vivo markers of the disease. For the N component, current suggested imaging biomarkers are brain atrophy, measured with magnetic resonance imaging (MRI), and cerebral hypometabolism measured with fluorodeoxyglucose-positron emission tomography (FDG-PET). Measurement of cerebral blood flow (CBF) using arterial spin labelling (ASL) MRI^4,5^ offers a non-invasive, non-ionising radiation alternative to FDG-PET in a dementia imaging protocol as CBF and metabolism are tightly coupled^6^. In AD, co-localisation of hypoperfusion and hypometabolism (measured with ASL-MRI and FDG-PET respectively) has been demonstrated in the posterior cingulate cortex (PCC), precuneus, angular gyrus and hippocampus^7–9^, with investigations using ASL-MRI alone reporting reduced perfusion in similar regions^10–14^.

Recently there is growing attention on vascular changes in dementia^2,15–18^, leading to the suggestion that a “V” component for measures of cerebrovascular dysfunction would be a useful addition to the existing AT(N) framework^17^. In an ASL study, the arterial transit time (ATT) is defined as the time taken for blood labelled in the feeding arteries to reach the microvasculature of the imaging volume. It is therefore a crucial timing parameter to consider when establishing ASL imaging protocols^19^. Physiologically, ATT may be an important measure of cerebrovascular health, since a longer ATT implies delayed delivery of oxygen and nutrients. Indeed, ATT is prolonged with increasing age^20,21^ and has been found to be lengthened in disease such as in Parkinson’s disease^22^. To date however, there is limited and conflicting literature on the association of prolonged ATT in AD. Yoshiura and colleagues found significantly reduced CBF in their AD cohort compared to controls, but no prolongation of ATT^14^, while a later study did report an increase in ATT in AD^12^, specifically in the left inferior frontal gyrus and middle cingulate gyrus. Both studies had limited sample sizes and do not provide information on ATT in the prodromal phase of dementia. Measuring ATT requires additional scan time and is not commonly employed in dementia protocols^23^. However, it has been demonstrated recently that the spatial coefficient of variation (sCoV) of ASL-CBF maps correlates well with independent measures of ATT^24,25^.

The utility of vascular imaging markers, such as sCoV, requires evidence of their sensitivity to gradations of cognitive decline that may precede development of dementia. Subjective cognitive decline (SCD)^26^ occurs prior to decline on formal neuropsychological tests, but recent imaging investigations suggest that changes are already evident beyond normal aging^27–29^ and may therefore represent a first step on the AD trajectory. When cognitive decline is evident on formal tests, as in mild cognitive impairment (MCI)^30,31^, memory is usually the first thing to be affected (amnestic MCI, aMCI). Cognitive changes involving memory as well as additional domains (multi-domain MCI, mMCI)^32^, may indicate further progression, before the loss of daily function that signals a clinical probable Alzheimer’s dementia diagnosis.

While previous work hints at a possible link between ATT and AD^12^, in MCI, sCoV was higher in temporal and total grey matter (indicating longer ATT) compared to that in controls, but did not differ from that in AD^33^. Conflicting results, heterogeneity of patient cohorts and the use of different imaging protocols make results to date difficult to interpret. To this end, we evaluated sCoV of CBF maps in a cohort comprising controls and individuals across the AD trajectory: SCD, MCI (single-domain aMCI and mMCI with amnestic component) and early probable AD. We hypothesised that sCoV of grey matter and temporal lobe CBF will increase monotonically between groups. We also hypothesised that since longer ATT implies delayed perfusion, sCoV of grey matter would correlate negatively with CBF.

## Results

**Figure 1:**
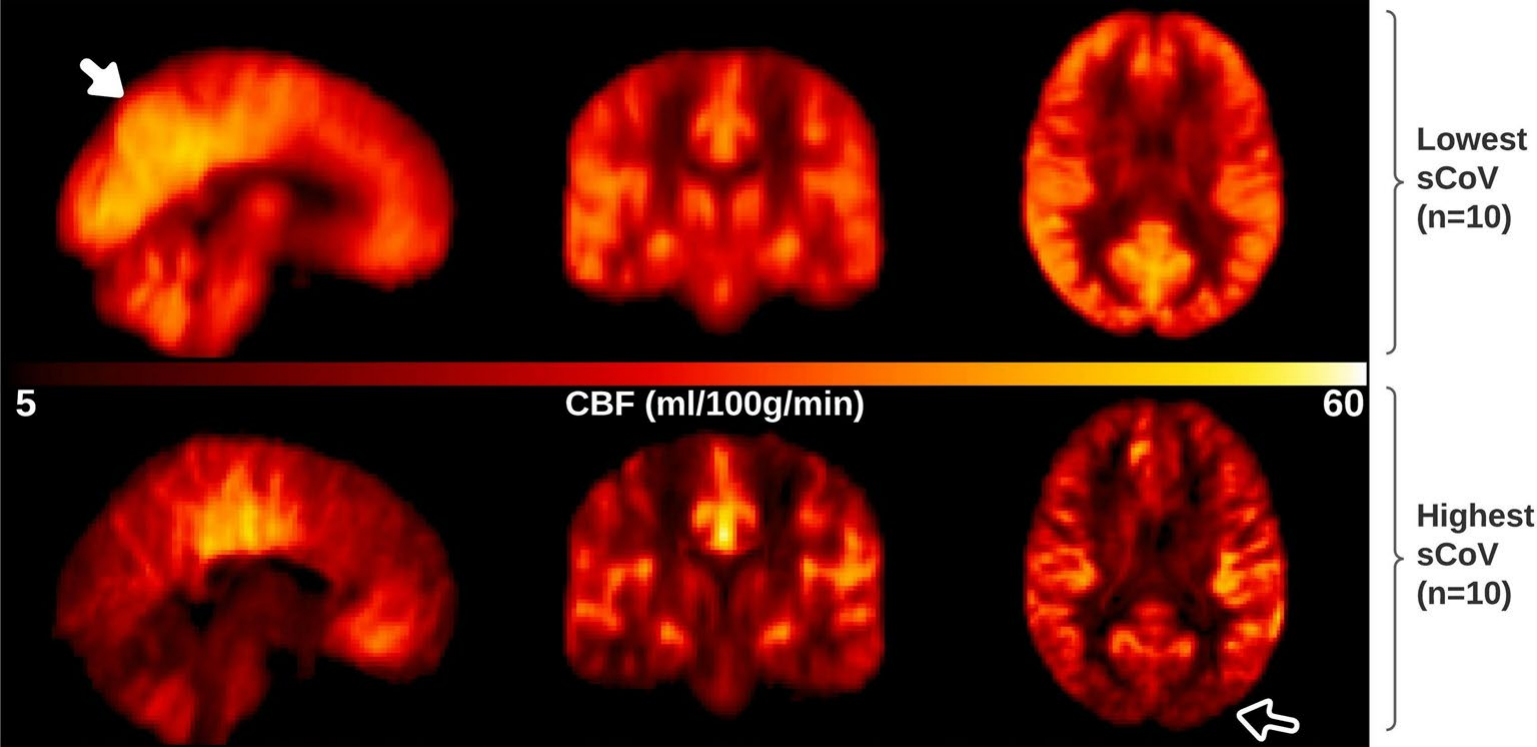
Illustrative figure depicting the appearance of CBF maps with low versus high sCoV in sagittal (left) coronal (middle) and axial (right) orientations. Top row, average non-PVC CBF maps for 10 participants with the lowest sCoV in GM ROI from the total cohort studied. This averaged CBF map representing low sCoV appears as expected, with high grey-white matter contrast, and increased perfusion in the region of the posterior cingulate cortex (PCC) and precuneus (see filled arrow). Bottom row, average non-PVC CBF maps for 10 participants with the highest sCoV in GM. The averaged CBF map for highest sCoV shows lower cortical perfusion overall, most notably in the posterior vascular territory (see open arrow), suggesting too short post labelling delay (PLD)/prolonged ATT.

Cognitive scores (Addenbrooke’s Cognitive Examination-III, ACE-III), age and distribution of sex were significantly different across groups (ANOVA, *χ*^*2*^, p < 0.05), with decreasing ACE-III scores from controls through to AD (see Table 1). Underlying vascular risk (assessed using aggregated scores for hypertension, dyslipidaemia, diabetes, and smoking), was not significantly different across groups. To visualise the appearance of CBF maps with low versus high sCoV, Fig. 1 shows averaged CBF maps, normalised to template space, for ten participants in the cohort with the lowest GM sCoV (range 36.3–41.1%, top row) and the ten participants with the highest sCoV (range 65.1–81.1%, bottom row). The lowest GM sCoV averaged CBF map appears a typical CBF map, with higher perfusion in grey matter compared to white matter, and increased perfusion in the region of the posterior cingulate cortex (PCC) and precuneus (see filled arrow, top left panel Fig. 1). The highest GM sCoV averaged CBF map has lower CBF overall, most notably in the posterior vascular territory (see open arrow bottom right panel Fig. 1), typical of ASL scans where the post labelling delay (PLD) is less than the ATT.

**Table 1:** Neuropsychological and demographic summary of participants.

	Controls n = 20	subjective cognitive decline (SCD) n = 44	Mild cognitive impairment (MCI) n = 45	Probable AD (AD) n = 13	Group statistic
ACE-III (SD)	94.6(3.8)	91.7(4.9)	85.2(7.1)	79.2(6.2)	F(3,118) = 27.4 p < 0.0001
Age (SD)	67.4(8.3)	69.0(7.7)	71.1(7.1)	74.9(6.2)	F(3,118) = 3.3 p = 0.024
Sex (% Female)	80	59	51	23	χ^2^ = 10.9, p = 0.012
Vascular risk factor ≥ 2 (%)	30	36	31	46	χ^2^ = 1.3, p = 0.739

Addenbrooke’s Cognitive Examination-III (ACE-III) and age are tabulated as group mean (standard deviation). ANOVA result comparing ACE-III and age, and a χ^2^ test comparing frequency distributions of sex and aggregated vascular risk factor across groups, is reported in the last column. Vascular risk factor is an aggregated measure for hypertension, dyslipidaemia, diabetes, and smoking, with a score of 1 for presence or treatment of each factor (range 0 to 4). Post-hoc pair-wise tests revelated a significant difference in age between controls and AD (p = 0.03). Comparing ACE-III between groups in post-hoc tests, significant differences were found for all pair-wise comparisons (p < 0.01), except for between control and SCD groups (p = 0.38).

Between-group differences of sCoV were tested using order-restricted ANCOVAs, controlling for age, sex, and site. We generated order restricted models (patterns of how sCoV may vary across disease severity groups) deemed to be plausible based on previous literature^12,14,33^ and combined MCI subtypes for statistical testing, resulting in four order restrictions (M_1_:Control < SCD < MCI < AD; M_2_:[Control = SCD] < MCI < AD; M_3_:Control < SCD < [MCI = AD]; M_4_:[Control = SCD] < [MCI = AD]). Additional order restriction models considering the aMCI and mMCI participants as separate groups and the associated results can be found in supplementary Sect. 2, along with a graphical representation of order restriction models. We used the Bayes factor (BF) as a statistical index of relative evidence for one (alternative) model of interest over another competing (null or alternative) model, e.g., ‘BF_10_’ represents the BF for the first order-restricted model M_1_ compared to the null hypothesis M_0_. A BF = 1 indicates that both models are equally as probable given the data, while the further the BF is from 1, the stronger the evidence is in favour of the model of interest (BF > 1), or the competing model (BF < 1).

**Figure 2:**
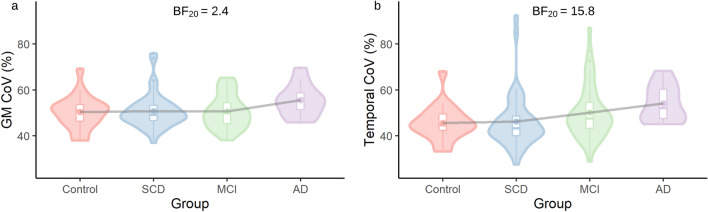
Spatial CoV group results (**a**) total grey matter (GM) and (**b**) temporal lobe ROIs in control group, subjective cognitive decline (SCD), mild cognitive impairment (MCI) and probable Alzheimer’s disease (AD). All data plotted are residual values after accounting for age, sex and site.

Figure 2 depicts total GM and temporal lobe sCoV for each group. In GM and the temporal lobe, model 2 (M_2_) was the preferred model (BF_20_ = 2.4 and 15.8 respectively), indicating that equal sCoV between the control and SCD groups and then an increase between SCD, MCI and AD groups best fit the data. In the frontal, parietal and occipital lobe ROIs, results favoured the null hypothesis (all BFs < 1, see Table 2) suggesting that there was no evidence for any differences in sCoV between groups in these regions.

**Table 2:** Bayes factors (BFs) for order-restricted sCOV models.

Bayes Factors	GM	Frontal	Parietal	Temporal	Occipital
BF_10_	1.6 [1.5]	0.2	0.2	11.6 [1.4]	0.3
BF_20_	**2.4**	0.3	0.4	**15.8**	0.9
BF_30_	0.3 [8.0]	0.1	0.1	6.0 [2.6]	0.2
BF_40_	0.5 [4.8]	0.2	0.2	3.9 [4.1]	0.2

BFs are expressed relative to the null model for total grey matter (GM), frontal, parietal, temporal occipital lobe ROIs. All BFs are corrected for age, sex and site. Bolded values indicate the strongest of the alternative models preferred over the null model (BFs > 1) for a given ROI; in both GM and temporal ROIs, this was M_2_: [Control = SCD] < MCI < AD. Values in square brackets are the BFs comparing the preferred alternative model directly to the other alternative models for that ROI. In frontal, parietal and occipital ROIs, results favoured the null model (all BFs < 1).

**Figure 3:**
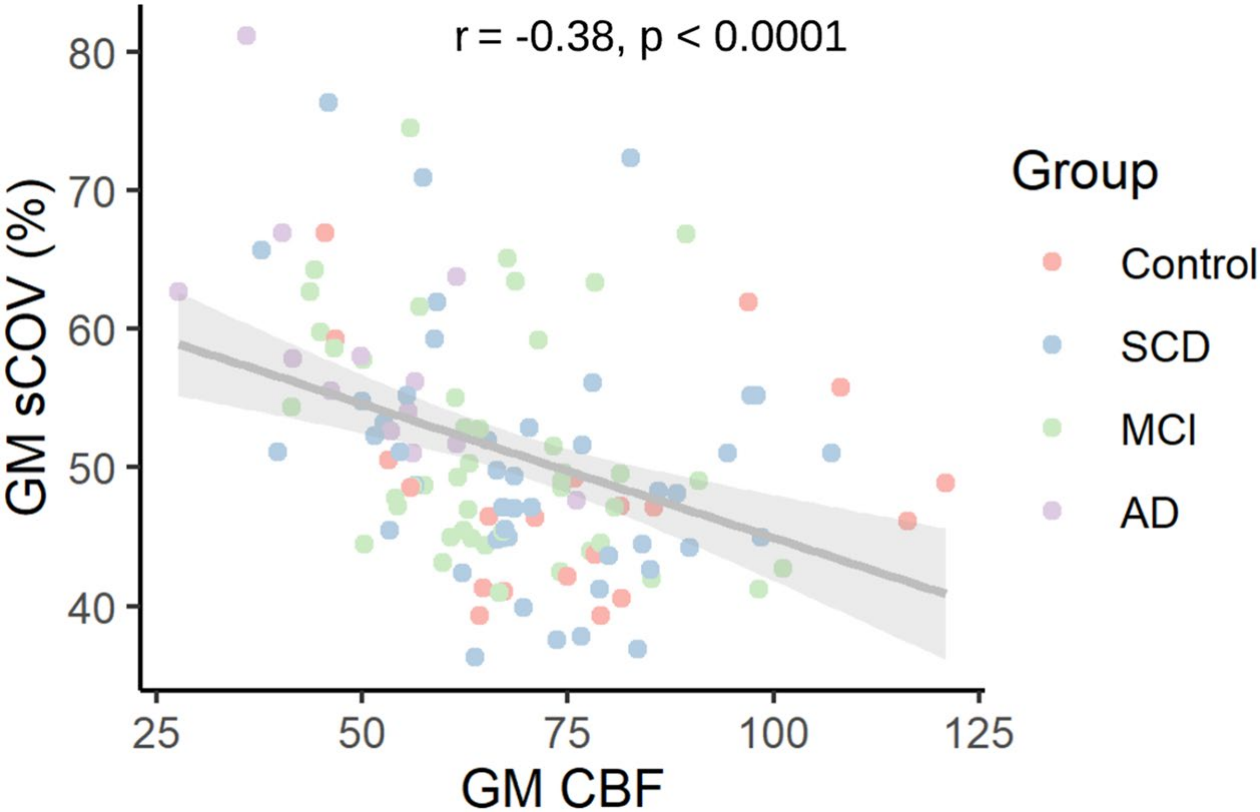
Spatial CoV (sCoV) and partial volume corrected cerebral blood flow (PVC CBF) in grey matter (GM) for each participant. Correlation between sCoV and CBF evaluated using Pearson’s correlation coefficient, with regression line and 95% confidence interval.

A statistically significant negative correlation was found between GM sCoV and GM CBF considering all participants (r = − 0.38, p = 1.5 × 10^–5^, see Fig. 3). No statistically significant correlations were found between sCoV and ACE-III, controlling for age, sex, and site, in total GM and the lobe ROIs, when tested either across or within groups (see https://osf.io/yfe5d/ for all results). Data from “travelling heads” (five non-study participants scanned at all three sites), showed that while individual CBF and sCoV were somewhat variable over time (see supplementary Figs. 2 and 3 respectively, panels a–e), group mean sCoV collected at the most recent time point were comparable across sites (panel f).

## Discussion

We found that sCoV increased between SCD, MCI and early probable AD groups in GM and in the temporal lobe. As expected, sCoV was negatively correlated with CBF. Overall, our results suggest that sCoV of ASL MRI may be a useful marker to monitor disease progression across the AD trajectory, and that vascular dysfunction (assessed here with a surrogate marker of ATT) could be a contributing factor.

To the best of our knowledge, there are only two studies measuring ATT directly in AD, and results to date are inconsistent^12,14^. Using sCoV to probe ATT effects indirectly, we found moderate evidence for an increasing sCoV in total GM and strong evidence for an increasing sCoV in the temporal lobe in the cognitively impaired, including probable AD dementia. In supplementary analyses examining single and multi-domain MCI subtypes separately, we were able to investigate sCoV in more subtle gradation of cognitive impairment. We found no difference in GM sCoV between controls and subjective complaints participants (both groups cognitively unimpaired on neuropsychological testing), but then a monotonic increase across aMCI, mMCI and AD groups. In the temporal lobe, a similar pattern was found, but with equal sCoV in the aMCI and mMCI groups, which may be unsurprising given both groups have significant memory impairment; the difference between the two groups comes from the involvement of other, additional cognitive domains in the mMCI group, likely relying on other circuits. Larger studies examining sCoV in single and multi-domain MCI are warranted.

Irrespective of MCI grouping, the increase we observed in sCoV in dementia differs from the study by Shirzadi and colleagues^33^, which found that sCoV only increased between cognitively unimpaired and MCI and *not* between MCI and AD. It could be expected that using different labelling methods (PASL in the ADNI cohort^33^ vs pCASL in the present work), might yield a different arterial proportion of the ASL signal and account for the difference in results between the two studies. In the current study we were guided by current best practice recommendations for perfusion ASL^19^ (a background suppressed, pCASL sequence with a 3D segmented readout), which should render our results more comparable to existing and new studies adopting these guidelines.

When using a single post-labelling delay (PLD) acquisition protocol, the effects of too short PLD (increased vascular component) and too long PLD (reduced SNR, due to T1 decay of the labelling bolus) need to be balanced. To this end, recommendations on what single PLD to use for different populations have been made^19^. Our cohort, with a mean age across all groups of 70.1 years straddles current guidelines of a 1.8 s PLD for < 70 years and 2.0 s for > 70 years. It is also recommended to use a PLD of 2.0 s for “adult clinical patient” populations^19^; however our cohort is a mix of cognitively normal and cognitively impaired participants. By using a PLD of 1.8 s rather than 2.0 s, we are better attuned to probe transit time effects with sCoV, since there will be a higher proportion of participants for which the labelled bolus at the time of imaging will be in feeding vessels, and not the capillary bed. A limitation should be noted, however, that in the regime of incomplete delivery of the labelled signal, CBF measurements may be less reliable than at longer PLDs, and by using a single PLD, reduced perfusion cannot be disentangled from transit time effects. Future studies collecting separate measurements of arterial transit time and CBF at longer PLDs are needed.

A higher sCoV due solely to transit time effects, for the reasons outlined above, is expected to yield a reduced CBF measurement. We find, as expected and previously demonstrated^24^, that sCoV and CBF are negatively correlated. In general, our GM CBF values measured in controls (see supplementary Table 2) of 42 ± 12 ml/100 g/min (group mean ± standard deviation) and 77 ± 21 ml/100 g/min after partial volume correction are within an expected range of 40–100 ml/100 g/min^19,34^ for GM perfusion. We also find that in regions shown previously to be sensitive to reductions in perfusion in cognitive decline, namely the posterior cingulate cortex (PCC), precuneus, angular gyrus and hippocampus^7,8,10,34,35^, a trend for decreasing perfusion between groups is present in our cohort (see supplementary Table 2), and persists after correcting for age, sex and site (see supplementary Table 3).

Spatial CoV is simple to compute and can be investigated retrospectively in previously acquired CBF maps in native or template space, and therefore may be easily adopted clinically. However, there are limitations to consider. By definition, sCoV is calculated over a region in space, and cannot be computed voxel-wise. In the current study we have examined total grey matter and lobar regions. Larger ROIs such as these include both proximal vessels and distal tissue, capturing the vascular distribution of the labelled signal and are well correlated with ATT^33^. With decreasing ROI size, e.g. smaller cortical regions, sCoV reflects more local heterogeneity of the CBF signal. This local heterogeneity will reflect somewhat the distribution of the label in microvascular and tissue compartments but with decreasing ROI size will be more impacted by measurement noise and have a less direct link to vascular dysfunction. A second limitation is that an increased sCoV is not specific to transit time effects and may be driven by other sources of CBF variation, such as motion and susceptibility related artefacts. In the current study, we took steps to mitigate this; after motion correction each participants CBF map was inspected, and we excluded a relatively high proportion of data (15%) based on other types of artefacts identified. A third limitation of the study is we do not have direct measurements of ATT with an independent method, such as collecting ASL data at multiple time-points^36^, or with flow-encoding^37^. However, a strong correlation with sCoV and an independent measure of ATT has been previously demonstrated^24^.

Extensive medical and clinical evaluation by experienced specialists (neurologists, gerontologists, psychiatrists) and neuropsychological testing was performed for each participant, along with an MRI scan incorporating T1-weighted, T2-weighted, FLAIR, and susceptibility-weighted imaging, interpreted by a neuroradiologist. Participants were then classified by consensus of the specialist multidisciplinary team. Since cognitive scoring is one element that contributes to clinical diagnosis, a significant difference between the groups in ACE-III scores (Table 1) was expected. However, the absence of significant associations between ACE-III with sCoV was unexpected. ACE-III scores, however, are coarse estimates of cognition, and are influenced by demographic factors and pre-morbid ability levels, all of which may have influenced the likelihood of detecting predicted associations in this sample^38,39^. Part of the medical evaluation included documenting hypertension, dyslipidaemia, diabetes and smoking, vascular factors known to increase the risk of developing AD^2,40^. In our cohort, there is a higher proportion of participants in the AD group with an aggregated vascular risk score above a threshold than the control participants (46% vs 30% respectively). However, the proportion was not statistically different when considering all groups. This may be due to the relatively small group size in our imaging study compared to larger epidemiological studies reporting vascular risk factors in AD^40,41^, and the lower odds ratios for vascular risk factors in MCI compared to AD^41^. While care was taken to exclude other neurological conditions other than probable Alzheimer’s Disease, participants in the current cohort have not, to date, been assessed with other methods making up the AT(N) framework such as amyloid or tau-PET, which would inform a biological diagnosis of Alzheimer’s disease. As such, our “AD” cohort, is better defined as probable Alzheimer’s dementia.

While CBF has been shown to have higher measurement variability than other imaging markers commonly used in AD^42^, sCoV is a ratio normalised to mean CBF, and may therefore be less variable. Nonetheless, see supplementary material for analysis of CBF variability due to physiological factors^43^ and steps taken to minimise this variability in our data. To compare variability in CBF and sCoV data collected across centres, five non-study, control participants were scanned at all three sites over the duration of the study data collection. In this “travelling head” data set, we found good agreement in group mean CBF and sCoV measured at each site (see Supplementary Figs. 2 and 3), providing evidence for consistent ASL acquisition and processing of data from all sites. It should be noted however, that our travelling heads with a mean age 40 years at the start of the study are younger than the study population. Given that CBF is likely to reduce with age^44^, and ATT increase with age^20^, the travelling head CBF and sCoV results are less relatable to the main study cohort, and hence we took a conservative approach and included site as a covariate in the statistical analysis.

In conclusion, our results provide evidence that the spatial heterogeneity of perfusion maps increase overall with cognitive decline in groups representing a path to dementia. Given previous work demonstrating strong correlation between spatial CoV and independent measures of ATT^24^, our results suggest that ATT, a measure of vascular health, is prolonged in cognitive decline. We also found that spatial CoV was negatively correlated with CBF, suggesting that delayed transit times contribute to reduced CBF measurements, in our own, and previous MCI and AD studies employing similar labelling parameters. Future investigations measuring CBF with a longer PLD, and ATT with an independent method are required to disentangle the separate contributions of perfusion and transit time effects, and will provide key information on whether cerebrovascular health is an overlooked factor in the aetiology of MCI and AD^17,18^.

## Methods

### Participants

MRI data from 122 participants enrolled in the longitudinal Brain Research New Zealand (BRNZ), Dementia Prevention Research Clinics (DPRCs) in Auckland, Christchurch and Dunedin were analysed. All participants met eligibility criteria: no significant history of psychiatric disorders, past or current alcohol problems, moderate-severe traumatic brain injury, pace-maker or neurological conditions other than mild probable AD. Participants underwent in-depth clinical, medical and neuropsychological assessments that included a minimum of two tests in each of five cognitive domains: verbal and visual memory, executive functioning, attention, visuospatial and language/verbal skills, as well as the Addenbrooke’s Cognitive Examination-III (ACE-III). Information about everyday cognitive functioning was obtained from an informant/relative. Cognitive impairment was defined as scores on two tests within a cognitive domain falling greater than one standard deviation below what is expected for their age and ability. Underlying vascular risk was assessed with an aggregated score considering hypertension, dyslipidaemia, diabetes and smoking. Scores were assigned using the following criteria: 1 each for hypertension, dyslipidaemia and diabetes if on current medication for the condition, or if not on treatment, confirmed by blood pressure/blood test result; 1 if a current or ex-smoker, where non-smokers (0 score) are those who never smoked, or smoked for less than 5 years and have been not smoking for 15 years or longer. From a range of 0 to 4, a threshold^45^ ≥ 2 was used as a measure overall vascular risk factor. MRI scans, including T1-weighted, T2-weighted, T2-FLAIR, and susceptibility-weighted images (SWI) were conducted and read clinically by a neuroradiologist to check for exclusionary conditions. Clinical diagnosis (guided by established criteria^46,47^) was decided by consensus of a specialist multidisciplinary team based on the clinical and neurological assessments, neuropsychology and clinical neuroimaging. Participants were classified into one of five groups: control older adults, SCD, aMCI (single domain), mMCI (multi-domain including memory) and early probable Alzheimer’s dementia. To directly compare imaging metrics collected at the three clinic sites, five additional non-study participants were scanned at all three sites (hereafter referred to as “travelling heads”; N = 5, 2 female), over a period of up to three years to cover the period of study data collection (see supplementary material for more information). The study procedure was approved by the national Health and Disability Ethics Committee. All participants (study and travelling heads) provided informed written consent before taking part in accordance with the New Zealand National Ethical Standards.

While a total of 153 scans were originally examined, 10 participants were excluded: four due to incidental findings (arachnoid cyst in the posterior fossa; large subdural haematoma with ongoing neurological deficits; acute thalamic infarct and tumour in right trigeminal cave), and six who had non-amnestic MCI. Of the 143 remaining, data from 21 participants were excluded due to image artefacts (e.g., poor labelling efficiency, registration errors, etc.), yielding a total of 122 participants (Auckland N = 91, Christchurch N = 16 and Dunedin N = 15). The number of participants classified into each cognitive status group and their demographics are provided in Table 1. No travelling head data was excluded.

### MRI acquisition

Imaging at all three centres was performed using MAGNETOM Skyra 3 T MRI scanners (Siemens Healthcare, Erlangen, Germany). In Auckland, data were collected using a 32-channel head coil, and in Dunedin and Christchurch, a 64-channel head and neck coil. All other imaging sequences and parameters were the same across the sites. Whole brain ASL images were acquired with currently recommended parameters^19^, namely a 3D gradient and spin echo (GRASE) readout and pseudo-continuous labelling (pCASL) prototype sequence, background suppression (four pulses), labelling duration = 1800 ms and single post-labelling delay = 1800 ms. Acquired voxel size was 3 × 3 × 4 mm, field of view = 192 × 192 × 168 mm, GRAPPA acceleration factor = 2, segments = 6, rBw = 2694 Hz/Px, TR/TE = 5000/14.34 ms, with each control-label pair repeated eight times and an M0 scan collected in-line (with the sequence default TR = 4 s) for a total scan duration = 8 min 31 s. A T1-weighted magnetisation-prepared rapid gradient-echo (MPRAGE) sequence, TR/TE/TI = 2000 ms/2.85 ms/880 ms, flip angle = 8 degrees, voxel size = 1 × 1 × 1 mm collected sagittally, with whole-brain coverage, was collected to aid tissue type segmentation and ASL image registration to a standard space. Additional clinically-oriented scans (T2, T2-FLAIR, and SWI) facilitated a clinical read. Travelling-heads participants were scanned with the same protocol, at approximately six-month intervals during the study period.

### Image processing

Full image processing details, including sample processing code are available in supplementary material and summarised here for brevity. DICOM images from all centres were converted to NifTi format, following Brain Imaging Data Structure (BIDS) conventions where specified (http://bids.neuroimaging.io/) at the time of analysis. Images were processed using FSL toolboxes, first *fsl_anat*, for tissue segmentation and to generate registration transformations of ASL images to Montreal Neurological Institute (MNI) space using the participants T1w image. Then *BASIL* (Bayesian Inference for Arterial Spin Labelling MRI)^48^, to compute motion corrected, partial volume corrected (PVC)^49^ and non-PVC CBF maps in MNI space. Magnetisation of arterial blood was computed voxel-wise using the acquired M0 image, corrected for T1 relaxation^19^. Calibrated perfusion values were calculated assuming a labelling efficiency of 60% (determined experimentally^50^), T1 blood at 3 T = 1.65 s, fixed bolus duration = 1.8 s and single compartment fitting^19^. Total grey matter (GM) PVC CBF was extracted using a binarised version (at 25% threshold) of the FSL GM tissue prior. PVC CBF in four brain regions previously shown to be sensitive to CBF changes in MCI and AD were extracted: the PCC, precuneus, hippocampus and angular gyrus.

The sCoV of ASL images^24^ was calculated as:

$$Spatial CoV=\frac{\sigma }{\mu }\times 100 \%$$

where $\sigma$ is the standard deviation of non-PVC CBF values within a ROI, and $\mu$ is the mean of non-PVC CBF values within the same ROI (since PVC would smooth the heterogenous ASL signal of interest). The same GM mask as described above was used as an ROI for sCoV analysis, along with the four lobes (bilateral masks). MRI data was collected and ASL images processed without knowledge of group classification.

### Statistical analysis

Global cognitive score (ACE-III) and age were compared across groups using ANOVAs, and a χ^2^ test was used to compare frequency distributions of sex and vascular risk factor across groups. A Bonferroni correction was applied to correct for multiple post-hoc pairwise tests when appropriate. Partial correlations of sCoV with global cognitive score, controlling for age, sex, and site, and correlations of sCoV with CBF were calculated using the psych package^51^ in R. We compared sCoV across groups using the BayesFactor^52^ package with default priors, and scripts adapted from^53^, available at https://osf.io/yfe5d/. Between-group differences of sCoV were tested using order-restricted ANCOVAs, controlling for age, sex, and site. Order restrictions^54,55^ enable direct comparisons of hypothesis-driven alternative models, rather than testing only the default hypothesis that all means differ (as per a conventional Bayesian ANOVA). For the purpose of statistical analysis, we combined the aMCI and mMCI participants into a single MCI group and generated order restricted models based on previous results^12,14,33^. Four order restrictions were tested (M_1_:Control < SCD < MCI < AD; M_2_:[Control = SCD] < MCI < AD; M_3_:Control < SCD < [MCI = AD]; M_4_:[Control = SCD] < [MCI = AD]). Order restriction models considering the aMCI and mMCI participants as separate groups were also tested (see supplementary Sect. 2).

Each order restricted (alternative) model was compared to the null hypothesis (M_0_ = no group differences). In the case of stronger evidence for an alternative model, it was then compared to the other order restrictions to evaluate differential fit of the other alternative models to the data. We used the Bayes factor (BF) as a statistical index of relative evidence for one (alternative) model over another competing (null or alternative) model (e.g., ‘BF_10_’ represents the BF for the first order-restricted model M_1_ compared to the null hypothesis M_0_, while ‘BF_12_’ represents the BF for the first order restricted model compared to the second). A BF of 1 is interpreted as no evidence for either of the models over the other; the further the BF moves away from 1, the stronger the evidence is in favour of either the model of interest (BF > 1), or the competing model (BF < 1). Typically a BF > 3 may be described as substantial evidence in favour of an alternative model over the null hypothesis^56,57^, while lower BFs are of interest when directly comparing two alternative models^58^, such as the order-restricted models we evaluate here.

## Supplementary Information

Supplementary Information.

## Data Availability

Complete results and analysis scripts are available at https://osf.io/yfe5d/. Data are available from the corresponding author on reasonable request.
