# Supplementary material for: Spatial variation of perfusion MRI reflects cognitive decline in mild cognitive impairment and early dementia
Source: Sci Rep. 2021 Dec 2;11:23325. doi: 10.1038/s41598-021-02313-z (PMC8639710; doi:10.1038/s41598-021-02313-z)
Supplement: Supplementary file 1 — Supplementary Information. [file 41598_2021_2313_MOESM1_ESM.pdf]

Spatial variation of perfusion MRI reflects cognitive decline in mild cognitive impairment and early dementia

Supplementary Information

1. Additional information to the main manuscript on the ASL processing method

- *fsl_anat* (https://fsl.fmrib.ox.ac.uk/fsl/fslwiki/fsl_anat) as distributed with FSL version 5.0.9 was used to process the T1w image (bias field correction; generate registration transformations of ASL images to Montreal Neurological Institute (MNI) space; brain extraction and tissue type segmentation);
- *Oxford_asl* (https://asl-docs.readthedocs.io/en/latest/oxford_asl.html) release version 3.9.17, was used to register the low resolution ASL images to standard space (using inputs from *fsl_anat*), compute magnetisation of arterial blood (*asl_calib*, voxel-wise using the supplied M0 image) and then compute the perfusion as described in[1] on a voxel wise basis using BASIL (Bayesian Inference for Arterial Spin Labelling MRI) toolbox.[2]

- *BASIL* options included were partial volume correction,[3] single compartment fitting,[1] fixed (not fitted) bolus duration = 1.8s, adaptive spatial smoothing of perfusion images and motion correction of ASL images using MCFLIRT.[4]

- Calibrated perfusion values were calculated using a labelling efficiency of 0.60 (determined experimentally as described in[5]), T1 blood at 3T = 1.65s.

- Sample processing code:
  $oxford_asl_software_vs -i $aslinput --iaf=ct --tis 3.60 --bolus 1.80 --casl --fslanat=$output_dir/"struc.anat" -c $M0input --tr 4.00 --cgain 10.00 --cmethod voxel --wp --t1b 1.65 --alpha 0.60 --fixbolus --spatial --mc --pvcorr --artoff -o $output_dir
  yielding non pvc and pvc maps in native (ASL), structural (not used) and standard (MNI) space.
- Calibrated perfusion was extracted from specific brain regions using Harvard-Oxford atlases[6] distributed with FSL, applied using *fslmaths* (https://fsl.fmrib.ox.ac.uk/fsl/fslwiki/Fslutils) and total cohort values extracted with Matlab (Mathworks, Natick, MA, USA) version R2015b.
- Specifically, the 25% probability threshold version of the Harvard-Oxford atlas was used, as on inspection, the atlas ROI borders more closely matched the structures in the template than the lower probability version, which extended outside the structures of interest and could therefore lead to partial volume effects.

2. Investigation of perfusion MRI in single and multi-domain mild cognitive impairment

INTRODUCTION: Being able to compare cerebral blood flow (CBF) and spatial coefficient of variation (sCoV) of CBF maps in normal aging, subjective cognitive decline (SCD), single domain amnestic MCI (aMCI), multi-domain MCI (mMCI) with amnestic component and probable Alzheimer's disease (AD), allows for an examination of specific patterns of group difference, representing the AD trajectory. For example, whether sCoV increases between all groups or in a more stepwise fashion is unknown (e.g., sCoV may be increased between SCD and aMCI, but not between aMCI and mMCI subtypes). We evaluated CBF and sCoV in five groups (C, SCD, aMCI, mMCI and AD). This analysis is additional to that presented in the main manuscript examining sCoV in a single MCI group combining the data from aMCI and mMCI participants.

METHOD: CBF analysis: In grey matter and four ROIs shown previously to be sensitive to change in MCI and AD, pvc CBF was compared between groups using the same analysis method described in the manuscript. Four order restriction models, deemed to be plausible based on previous literature, were tested (see middle row, supplementary figure 1):

M1:Control>SCD>aMCI>mMCI>AD;

M2:[Control=SCD]>aMCI>mMCI>AD;

M3:Control>SCD>[aMCI=mMCI]>AD;

M4:[Control=SCD]>[aMCI=mMCI]>AD.

These models were devised, because there exists a sizeable body of evidence[7–14] that CBF decreases in both MCI and AD relative to controls and AD compared to MCI. Therefore, in the models above, SCD > aMCI and mMCI > AD was fixed, because we believe there is strong evidence to support this. What was varied, were the modelled pattern of CBF changes between Control and SCD groups, and aMCI and mMCI groups since CBF in the aforementioned groups has been less well studied.

sCoV analysis: In this case, there is less and conflicting literature[15–17] to inform model selection. Previous results may be summarised as follows: for sCoV, C < MCI = AD [17], for ATT: C = AD [16] and C < AD [15]. We therefore tested the same order restrictions as CBF above (albeit with sCoV *increasing* with disease severity), **plus** four that did not only assume increasing sCoV in AD relative to mMCI, but also modelled equality between mMCI and AD, namely (see bottom row, supplementary figure 1):

M5:Control<SCD<aMCI<[mMCI=AD];

M6:[Control=SCD]<aMCI<[mMCI=AD];

M7:Control<SCD<[aMCI=mMCI=AD];

M8:[Control=SCD]<[aMCI=mMCI=AD].

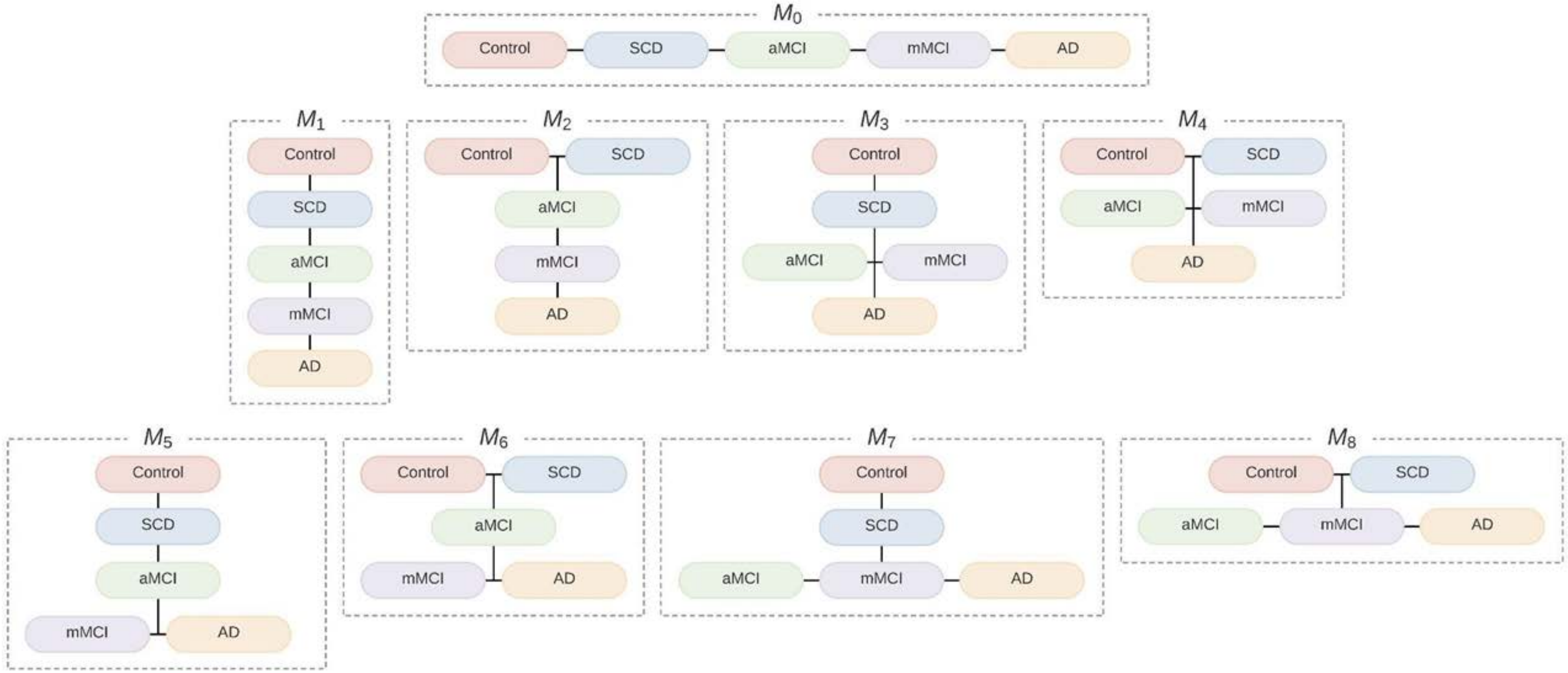

Supplementary Figure 1*. *Order restriction models. *Top row, schematic of null hypothesis (M_0), middle row, order-restricted models (M_1 to M_4), tested with respect to pvc CBF in control group, subjective cognitive decline (SCD), amnestic MCI (aMCI), multi-domain MCI (mMCI) and probable Alzheimer's disease (AD). Bottom row, additional order-restricted models (M_5 to M_8) tested with respect to sCoV in the same groups. Figure was created using Lucid Charts (https://www.lucidchart.com/), 2019.*

RESULTS:

	controls n=20	subjective cognitive decline (SCD) n=44	amnestic MCI (aMCI) n=27	multi-domain MCI (mMCI) n=18	probable AD (AD) n=13	group statistic
ACE-III (SD)	94.6(3.8)	91.7(4.9)	86.6(6.4) **,†	83.2(7.9) **,††	79.2(6.2) **,††,‡	F(4,117)= 22.0 p<0.0001
Age (SD)	67.4(8.3)	69.0(7.7)	70.4(8.2)	72.1(5.2)	74.9(6.2)	F(4,117)= 2.6 p=0.041
Sex (% Female)	80	59	48	56	23	χ^2=11.2, p=0.024
Vascular risk factor ≥ 2 (%)	30	36	26	39	46	χ^2=2.1, p=0.72

***Supplementary Table 1. Neuropsychological and demographic summary of participants**. ACE-III and age are tabulated as group mean (standard deviation). ANOVA result comparing ACE-III and age, and a χ^2 test comparing frequency distributions of sex and aggregated vascular risk factor across groups, is reported in the last column. Vascular risk factor is an aggregated measure for hypertension, dyslipidaemia, diabetes, and smoking, with a score of 1 for presence or treatment of each factor (range 0 to 4). Post-hoc tests were performed using Bonferroni's multiple comparison test. There were no significant differences in age between any two groups in post-hoc tests. Comparing ACE-III between groups in post-hoc tests, significant differences were found comparing controls vs. aMCI, controls vs. mMCI, controls vs. early AD (represented as **p<0.0001); between SCD and aMCI, SCD vs. mMCI and SCD vs. AD (represented as †p<0.01, ††p<0.0001); and comparing aMCI and AD groups (represented ‡p<0.01). Abbreviation: ACE-III - Addenbrooke's Cognitive Examination-III.*

	controls	subjective cognitive decline	amnestic MCI	multi-domain MCI	probable AD
**Non-PVC CBF**	20	44	27	18	13
Total GM CBF	41.6	38.0	36.6	33.0	26.3
Total GM SD	11.8	8.9	8.4	6.1	6.1
PCC CBF	63.6	59.4	56.3	52.3	42.8
PCC CBF SD	18.8	14.4	14.7	10.0	9.4
Precuneus CBF	55.7	51.8	49.1	44.4	34.5
Precuneus CBF SD	18.3	12.6	14.8	9.5	8.1
Hippocampus CBF	49.7	47.3	40.2	39.8	34.6
Hippocampus CBF SD	15.1	12.3	9.4	8.6	8.9
Angular Gyrus CBF	54.5	49.3	49.1	43.4	36.7
Angular Gyrus CBF SD	13.5	10.5	11.6	9.6	8.9
**PVC CBF**					
Total GM pvc CBF	76.6	70.6	68.5	63.1	50.9
Total GM pvc SD	21.1	16.3	15.1	12.3	12.7
PCC pvc CBF	104.0	97.6	94.4	89.3	71.4
PCC pvc CBF SD	30.1	23.6	21.8	16.8	16.9
Precuneus pvc CBF	97.5	91.1	88.2	81.5	62.5
Precuneus pvc CBF SD	31.2	22.7	23.3	18.5	15.7
Hippocampus pvc CBF	102.0	99.1	88.1	89.2	79.3
Hippocampus pvc CBF SD	28.8	25.1	20.1	21.5	20.4
Angular Gyrus pvc CBF	96.2	87.5	87.2	81.2	67.2
Angular Gyrus pvc CBF SD	24.0	19.2	20.5	18.6	16.6
**SCOV**					
Total GM sCOV	48.1	50.2	49.4	54.3	58.4
Total sCOV SD	7.6	9.0	7.7	8.2	8.7
Frontal sCoV	60.4	62.9	58.7	66.6	68.2
Frontal sCoV SD	9.7	10.9	10.1	11.2	9.8
Parietal sCoV	49.1	50.0	47.5	54.3	56.9
Parietal sCoV SD	10.3	10.1	10.2	9.6	12.3
Temporal sCoV	42.8	45.5	50.6	51.7	57.9
Temporal sCoV SD	7.7	13.1	14.1	10.8	9.3
Occipital sCoV	40.0	40.2	41.5	45.1	50.1
Occipital sCoV SD	13.4	11.3	13.1	10.6	9.8

***Supplementary Table 2. CBF and sCoV results**. CBF [ml/100g/min], partial volume corrected (pvc) CBF [ml/100g/min], pvc ratio (pvc CBF/CBF), spatial coefficient of variation (sCoV) [%] and standard deviation (SD) across the group for each variable analysed. Values are uncorrected for age, sex and site.*

Bayes factors	GM	PCC	Precuneus	Angular gyrus	Hippocampus
BF_{10}	**63.4**	**31.1**	**25.3**	**12.3**	7.8 [2.0]
BF_{20}	37.0 [1.7]	21.1 [1.5]	23.8 [1.1]	6.1 [2.0]	9.8 [1.6]
BF_{30}	45.2 [1.4]	29.3 [1.1]	20.5 [1.2]	10.8 [1.1]	12.7 [1.2]
BF_{40}	28.9 [2.2]	23.7 [1.3]	21.3 [1.2]	5.8 [2.1]	**15.5**

***Supplementary Table 3. Bayes factors (BFs) for order-restricted models of pvc CBF**. BFs are expressed relative to the null hypothesis (BF_{10} is the BF for M_1 compared to the null) in total grey matter (GM), posterior cingulate cortex (PCC), precuneus, angular gyrus and hippocampus ROIs. Results are corrected for age, sex and site. Bolded values indicate the strongest of the alternative models preferred over the null model (BFs > 1); for the Hippocampus, M4:[Control=SCD]>[aMCI=mMCI]>AD, and for all other ROIs, M1:Control>SCD>aMCI>mMCI>AD. Values in square brackets are the BFs comparing the preferred alternative model directly to the other alternative models for that ROI.*

Bayes Factors	GM	Frontal	Parietal	Temporal	Occipital
BF_{10}	4.8 [1.3]	0.4	0.4	10.6 [1.5]	0.4
BF_{20}	**6.3**	0.6	0.7	12.9 [1.2]	0.8
BF_{30}	1.6 [3.9]	0.2	0.2	11.6 [1.4]	0.3
BF_{40}	2.4 [2.6]	0.3	0.4	**15.8**	0.9
BF_{50}	2.1 [3.0]	0.4	0.3	7.3 [2.2]	0.3
BF_{60}	3.7 [1.7]	0.6	0.7	9.1 [1.7]	0.7
BF_{70}	0.3 [21.0]	0.1	0.1	6.0 [2.6]	0.2
BF_{80}	0.5 [12.6]	0.2	0.2	3.9 [4.1]	0.2

***Supplementary Table 4. Bayes factors (BFs) for order-restricted models of sCOV.** BFs are expressed relative to the null in the total grey matter (GM), frontal, parietal, temporal occipital lobe ROIs. Results are corrected for age, sex and site. Bolded values are indicate the strongest of the alternative models preferred relative to the null model (BF > 1); for GM, M2:[Control = SCD]<aMCI<mMCI<AD, and for the Temporal ROI, M4:[Control=SCD]<[aMCI=mMCI]<AD. Values in square brackets are the BFs comparing the preferred alternative model directly to the other alternative models for that ROI. In all other ROIs, results favoured the null model (all BFs < 1).*

CONCLUSION: In this study we investigated how cerebral perfusion (partial volume corrected ASL MRI; CBF), and spatial coefficient of variation of the perfusion maps (sCoV), varied across control, SCD, aMCI, mMCI and early AD groups. CBF decreased linearly across all groups when ordered by disease severity in GM, PCC, precuneus and angular gyrus, but in a more stepwise fashion (an overall decrease, but not a decrease between each group) in the hippocampus. sCoV increased between SCD, aMCI, mMCI and early AD groups in GM, and in stepwise

manner in the temporal lobe. Overall, our results indicate a reduction in CBF measured with ASL that may be able to monitor disease progression across the AD trajectory, and hint that vascular dysfunction (assessed here with a surrogate marker, sCoV) could be a contributing factor.

3. Between site scanner reproducibility

INTRODUCTION: To directly compare imaging metrics collected at the three clinic sites, Auckland, Dunedin and Christchurch, five non-study participants were scanned at all three sites, herein referred to as the “travelling heads”.

METHOD: The travelling head data set consisted of 5 subjects (2 female) mean age and standard deviation at time of first scan 40±7 years, scanned at each study site, over a period of up to three years to cover the same period as the main study data collection. Dunedin and Christchurch clinics had a later start to data collection and hence why traveling head data was not collected over the same period as in Auckland. CBF maps were calculated in the same way as described above, but values were extracted from a grey matter mask (generated by *oxford_asl*) in native space to minimise any small alterations in CBF that may arise due to registration to standard space in this small sample.

RESULTS: Individual CBF and sCoV are shown in supplementary figures 1 and 2 respectively (panels A to E for each participant). Mean ± standard deviation across the group CBF at the most recent time point (panel F) was 49.1 ± 13.2 ml/100g/min, 49.1 ± 8.6 ml/100g/min and 48.1 ± 2.8 ml/100g/min and sCoV was 43.0 ± 5.3%, 38.1 ± 7.8% and 45.7±5.6%, in Auckland, Christchurch and Dunedin respectively.

DISCUSSION: We do not have a large enough sample or number of repeated scans to perform valid statistical tests to assess variance between centres and over time (indeed the number of subjects needed would be prohibitively large).[18] Rather, perfusion and sCoV values for all subjects were plotted, to relate the magnitude of differences in measured values in the same subject across centres to differences in the same subjects across time.

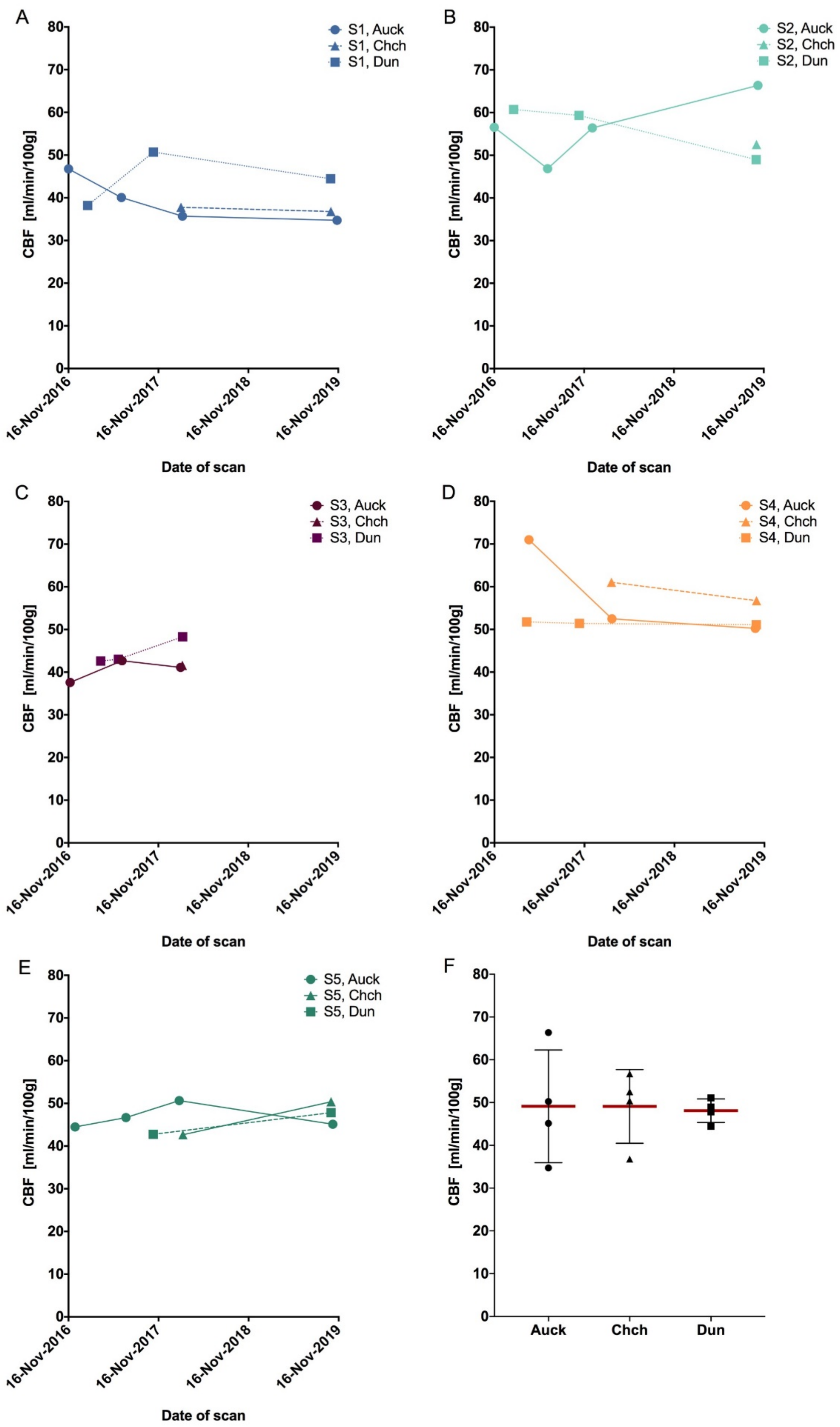

Supplementary Figure 2: *Individual CBF (panels A to E for each participant) and mean CBF across travelling head participants at the latest time point (panel F) for Auckland Christchurch and Dunedin. Error bars in F are +/- 1 standard deviation.*

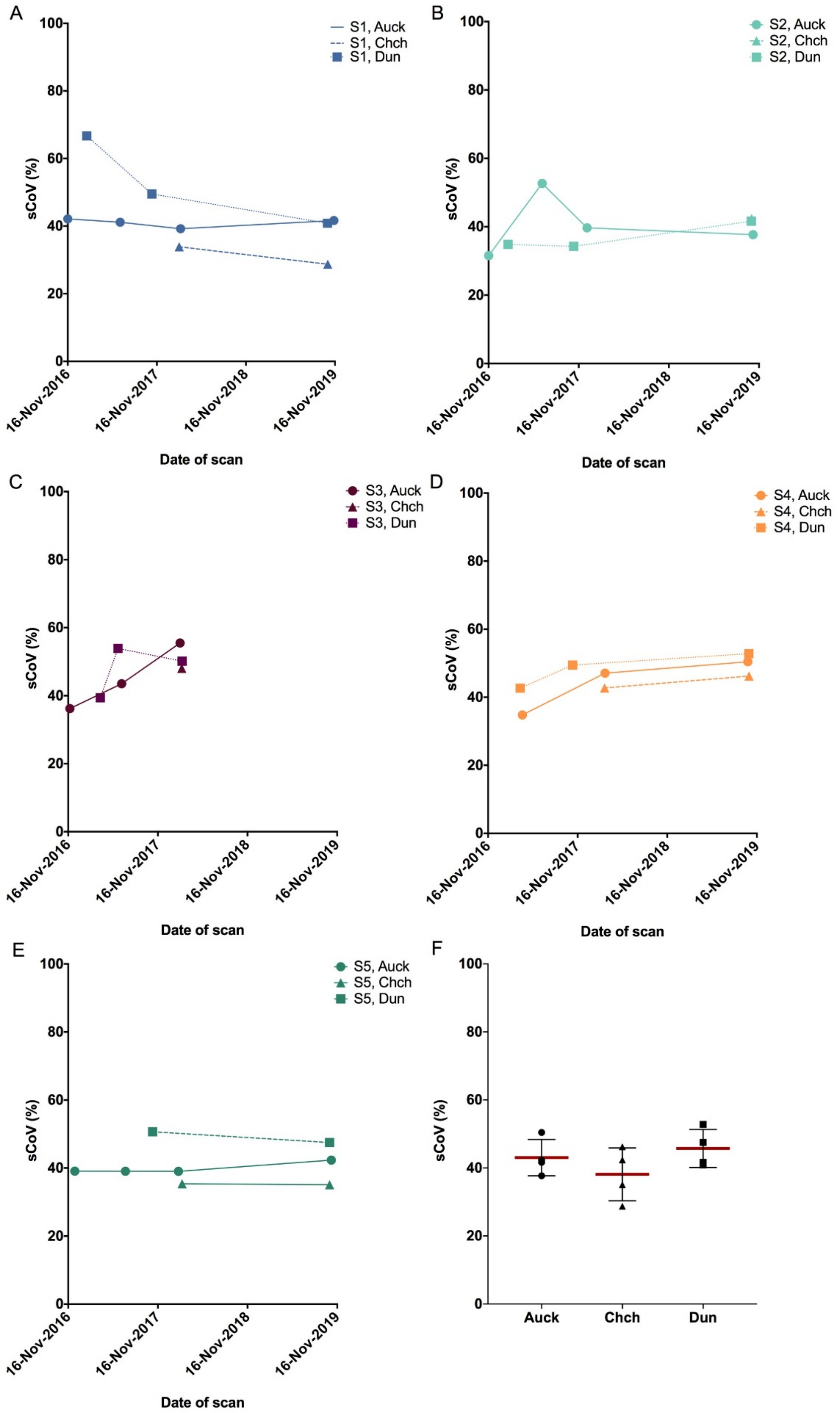

Supplementary Figure 3*. Individual spatial CoV (panels A to E for each participant) and mean value across travelling head participants at latest time point (panel F) for Auckland Christchurch and Dunedin. Error bars in F are +/- 1 standard deviation.*

4. Investigation of additional modifiable covariates that may modify perfusion results

BACKGROUND: While a multitude of factors have been reported that modify perfusion values, such as age and sex, the majority of literature to date have not accounted for other, possibly modifiable moderators on CBF such as caffeine, nicotine, sleep and diurnal effects (see[19] for a more complete list and excellent review). In our study, we corrected for non-modifiable factors age, sex and site in the statistical analysis. In addition, we took steps to control for some of the modifiable moderators as recommended in.[19] Namely, 1) recording acute caffeine and nicotine intake, 2) instructing participants to keep their eyes closed (to avoid visual stimulation) but stay awake during the scan and then asking for an awakeness rating in situ immediately post-scan to determine whether they did fall asleep, and 3) where possible, scanning at the same time of day. We purposefully didn't ask people to abstain from caffeine before the scan (so as not to induce withdrawal effects)[19], and instead recorded information about caffeine intake. In this supplementary analysis we sought to investigate how these additional covariates may impact our results. However, the aforementioned steps 1) to 3) were not performed for some participants at the commencement of the study, therefore this investigation only includes a subset of the main study data.

METHODS: The supplementary analysis was performed in a sub-sample of 81 subjects (out of full sample 122) where steps 1) to 3) above were taken. An arbitrary cut off 3 for the awakeness rating was used to binarise the variable ('asleep': <3; 'awake': ≥ 3). Caffeinated drinks consumed within approximately 75 minutes of the ASL scan were considered acute (yielding the greatest plasma caffeine levels[20] and caffeine induced reduction in CBF[21]). None of the participants in the subset of data was a smoker; this variable was therefore not included as additional covariate. We compared a model including all covariates (age, sex, site, acute caffeine and binary awakeness) to one that included just the three covariates from the main study analysis (age, sex, and site). If the two additional covariates play an important role in predicting the outcome variables (i.e., if they explain any additional variance), the model that includes the additional variables should be stronger than the original model, and therefore the BF should be greater than 1.

RESULTS: Including acute caffeine and binary awakeness as additional covariates in the models did not improve any of the CBF models (GM: BF = 0.21; PCC: BF = 0.26; Precuneus: BF = 0.24; HC: BF = 0.27; Angular gyrus: BF = 0.20). For sCOV, the results were similar (GM: = 0.76; frontal lobe: BF = 0.38; parietal lobe: BF = 0.38; occipital lobe: BF = 0.27), but there was less conclusive evidence for the temporal lobe (BF = 1.13). See https://osf.io/yfe5d/ for all results and analysis scripts.

DISCUSSION: Our results suggest that acute caffeine intake and subjective awakeness during the scans did not play a role in the reported results. However, we did not have this data in our full study cohort. Furthermore, while we took a simple approach of asking participants to remain awake and self-report after the scan, this is subjective and ideally, assessment of sleep (e.g., via simultaneous EEG) would be performed to try and quantify more objectively the

effect of sleep on cerebral perfusion. The influence of sleep on perfusion measurements is further complicated by the type and timing of sleep; since non-REM sleep appears to have a decreasing perfusion effect, vs REM sleep which has minimal effect, and being awoken from sleep or awake spontaneously seems to have opposing effects on perfusion [19].

Supplementary References

1. Alsop DC, Detre JA, Golay X, et al. Recommended implementation of arterial spin-labeled perfusion MRI for clinical applications: A consensus of the ISMRM perfusion study group and the European consortium for ASL in dementia. *Magn Reson Med* 2015; 73: 102–116.

2. Chappell MA, Groves AR, Whitcher B, et al. Variational Bayesian Inference for a Nonlinear Forward Model. *Trans Sig Proc* 2009; 57: 223–236.

3. Chappell MA, Groves AR, MacIntosh BJ, et al. Partial volume correction of multiple inversion time arterial spin labeling MRI data. *Magn Reson Med* 2011; 65: 1173–1183.

4. Jenkinson M, Bannister P, Brady M, et al. Improved optimization for the robust and accurate linear registration and motion correction of brain images. *Neuroimage* 2002; 17: 825–841.

5. Vidorreta M, Wang Z, Rodríguez I, et al. Comparison of 2D and 3D single-shot ASL perfusion fMRI sequences. *NeuroImage* 2013; 66: 662–671.

6. Desikan RS, Ségonne F, Fischl B, et al. An automated labeling system for subdividing the human cerebral cortex on MRI scans into gyral based regions of interest. *NeuroImage* 2006; 31: 968–980.

7. Chen Y, Wolk DA, Reddin JS, et al. Voxel-level comparison of arterial spin-labeled perfusion MRI and FDG-PET in Alzheimer disease. *Neurology* 2011; 77: 1977–1985.

8. Dolui S, Vidorreta M, Wang Z, et al. Comparison of PASL, PCASL, and background-suppressed 3D PCASL in mild cognitive impairment. *Hum Brain Mapp* 2017; 38: 5260–5273.

9. Binnewijzend MAA, Kuijer JPA, Benedictus MR, et al. Cerebral Blood Flow Measured with 3D Pseudocontinuous Arterial Spin-labeling MR Imaging in Alzheimer Disease and Mild Cognitive Impairment: A Marker for Disease Severity. *Radiology* 2013; 267: 221–230.

10. Collij LE, Heeman F, Kuijer JPA, et al. Application of Machine Learning to Arterial Spin Labeling in Mild Cognitive Impairment and Alzheimer Disease. *Radiology* 2016; 281: 865–875.

11. Dai W, Lopez OL, Carmichael OT, et al. Mild Cognitive Impairment and Alzheimer Disease: Patterns of Altered Cerebral Blood Flow at MR Imaging. *Radiology* 2009; 250: 856–866.

12. Johnson NA, Jahng G-H, Weiner MW, et al. Pattern of Cerebral Hypoperfusion in Alzheimer Disease and Mild Cognitive Impairment Measured with Arterial Spin-labeling MR Imaging: Initial Experience. *Radiology* 2005; 234: 851–859.

13. Kim SM, Kim MJ, Rhee HY, et al. Regional cerebral perfusion in patients with Alzheimer's disease and mild cognitive impairment: effect of APOE Epsilon4 allele. *Neuroradiology* 2013; 55: 25–34.

14. Alsop DC, Detre JA, Grossman M. Assessment of cerebral blood flow in Alzheimer's disease by spin-labeled magnetic resonance imaging. *Ann Neurol* 2000; 47: 93–100.

15. Mak HKF, Chan Q, Zhang Z, et al. Quantitative Assessment of Cerebral Hemodynamic Parameters by QUASAR Arterial Spin Labeling in Alzheimer's Disease and Cognitively Normal Elderly Adults at 3-Tesla. *J Alzheimers Dis* 2012; 31: 33–44.

16. Yoshiura T, Hiwatashi A, Yamashita K, et al. Simultaneous Measurement of Arterial Transit Time, Arterial Blood Volume, and Cerebral Blood Flow Using Arterial Spin-Labeling in Patients with Alzheimer Disease. *Am J Neuroradiol* 2009; 30: 1388–1393.

17. Shirzadi Z, Stefanovic B, Mutsaerts HJMM, et al. Classifying cognitive impairment based on the spatial heterogeneity of cerebral blood flow images: ASL-CBF Spatial Heterogeneity in AD. *J Magn Reson Imaging*. Epub ahead of print 21 January 2019. DOI: 10.1002/jmri.26650.

18. Melzer TR, Keenan RJ, Leeper GJ, et al. Test-retest reliability and sample size estimates after MRI scanner relocation. *NeuroImage* 2020; 211: 116608.

19. Clement P, Mutsaerts H-J, Václavů L, et al. Variability of physiological brain perfusion in healthy subjects – A systematic review of modifiers. Considerations for multi-center ASL studies. *J Cereb Blood Flow Metab* 2018; 38: 1418–1437.

20. Lelo A, Birkett DJ, Robson RA, et al. Comparative pharmacokinetics of caffeine and its primary demethylated metabolites paraxanthine, theobromine and theophylline in man. *Br J Clin Pharmacol* 1986; 22: 177–182.

21. Vidyasagar R, Greyling A, Draijer R, et al. The effect of black tea and caffeine on regional cerebral blood flow measured with arterial spin labeling. *J Cereb Blood Flow Metab Off J Int Soc Cereb Blood Flow Metab* 2013; 33: 963–968.
